# Palopegteriparatide for adults with chronic hypoparathyroidism: skeletal dynamics through 3 years of the phase 2 PaTH forward trial

**DOI:** 10.1093/jbmr/zjag013

**Published:** 2026-02-04

**Authors:** Mishaela R Rubin, Bart L Clarke, Lorenz C Hofbauer, Aliya Khan, Peter Schwarz, Tamara Vokes, Intekhab Ahmed, Andrea Palermo, Filomena Cetani, Uberto Pagotto, Carol Zhao, Michael S Ominsky, Bryant Lai, Jenny Ukena, Aimee D Shu, Lars Rejnmark

**Affiliations:** Division of Endocrinology, Columbia University, New York, NY 10032, United States; Division of Endocrinology, Mayo Clinic, Rochester, MN 55905, United States; Division of Endocrinology, Diabetes, and Metabolic Bone Diseases, Technische Universität Dresden Medical Center, Dresden, 01307, Germany; Division of Endocrinology, Metabolism, and Geriatrics, McMaster University, Hamilton, ON L8N 3Z5, Canada; Department of Internal Medicine and Endocrinology, Rigshospitalet, Copenhagen, DK-2100, Denmark; Division of Endocrinology, Diabetes, and Metabolism, University of Chicago, Chicago, IL 60637, United States; Thomas Jefferson University, Philadelphia, PA 19144, United States; Unit of Metabolic Bone and Thyroid Disorders, Fondazione Policlinico Campus Bio-Medico, Rome, 00128, Italy; Unit of Endocrinology and Diabetes, Campus Bio-Medico University, Rome, 00128, Italy; Department of Clinical and Experimental Medicine, Endocrine Unit, University of Pisa, Pisa, 56124, Italy; Division of Endocrinology and Diabetes Prevention and Care, IRCCS Azienda Ospedaliero-Universitaria di Bologna, Department of Medical and Surgical Sciences (DIMEC), Alma Mater Studiorum University of Bologna, Bologna, 40126, Italy; Endocrine and Rare Disease, Medical Sciences, Ascendis Pharma Inc., Palo Alto, CA 94304, United States; Endocrine and Rare Disease, Medical Sciences, Ascendis Pharma Inc., Palo Alto, CA 94304, United States; Endocrine and Rare Disease, Medical Sciences, Ascendis Pharma Inc., Palo Alto, CA 94304, United States; Endocrine and Rare Disease, Medical Sciences, Ascendis Pharma Inc., Palo Alto, CA 94304, United States; Endocrine and Rare Disease, Medical Sciences, Ascendis Pharma Inc., Palo Alto, CA 94304, United States; Department of Clinical Medicine and Endocrinology, Aarhus University Hospital, Aarhus, 8200, Denmark

**Keywords:** parathyroid-related disorders, hormone replacement/receptor modulators, biochemical markers of bone turnover, PTH/Vit D/FGF23, clinical trials

## Abstract

Hypoparathyroidism is an endocrine disease caused by insufficient levels of PTH, which acts directly on bone and kidney and indirectly on the intestine to regulate calcium and phosphate balance. In clinical trials, palopegteriparatide (TransCon PTH) treatment enabled independence from conventional therapy (no active vitamin D, ≤600 mg/d calcium) and maintained serum biochemistries within normal ranges. The current analyses describe patterns of change in BMD, serum bone turnover markers, and serum and urine calcium in adults with chronic hypoparathyroidism treated with palopegteriparatide through 3 years of the PaTH Forward trial. Baseline BMD Z-scores for the LS, TH, FN, and 1/3 distal radius were above zero, indicating bone mass exceeding age-adjusted normative values. BMD decreased from these elevated baseline levels with palopegteriparatide treatment, with larger reductions during the first 26 weeks and modest declines thereafter. Mean BMD Z-scores at week 162 remained above zero for all 4 sites. Participants with lower baseline BMD (Z-scores below −1 and T-scores below −2.5) generally exhibited lesser declines in BMD versus those with higher baseline BMD. Palopegteriparatide treatment was associated with early increases in bone resorption (serum C-terminal telopeptide of type I collagen, CTx) and bone formation (serum procollagen type 1 N-terminal propeptide, P1NP) that peaked at weeks 12 and 26, respectively, followed by declines to levels moderately higher than baseline at week 162. Mean CTx and P1NP in the overall population and the subgroup of postmenopausal women were below their upper limits of normal from weeks 58-162. At week 162, mean serum and median urine calcium remained within normal ranges and 91% of participants were independent from conventional therapy. These results suggest that long-term palopegteriparatide therapy in adults with chronic hypoparathyroidism gradually returns the skeleton toward its natural state thereby enhancing the skeleton’s contribution to calcium homeostasis.

## Introduction

Hypoparathyroidism is an endocrine disease caused by insufficient levels of PTH. Under normal physiological conditions, PTH is the primary regulator of the calcium/phosphate balance by promoting renal calcium reabsorption, increasing bone remodeling, and stimulating renal expression of 1α-hydroxylase to generate active vitamin D (calcitriol) that promotes intestinal calcium absorption.^1^^,^^2^ In the absence of sufficient PTH, patients develop hypocalcemia and elevations in urinary fractional excretion of calcium.^2^ PTH also promotes renal phosphate excretion, and individuals with hypoparathyroidism are thus prone to hyperphosphatemia.^1^^,^^3^ Chronic hypoparathyroidism is associated with low bone remodeling that predisposes to increased BMD, including at the spine, hip, and FN.^4–8^ Reduced bone remodeling also raises potential concerns regarding bone quality, with decreased bone matrix renewal leading to increased matrix mineralization density.^4^^,^^5^^,^^9–11^ Bone in individuals with hypoparathyroidism demonstrates inferior material strength properties that may reduce its resistance to microfractures.^11^^,^^12^ Low bone remodeling can also have metabolic consequences, including a reduced ability to buffer calcium and phosphate loads due to reduced osteoid.^13^^,^^14^

Conventional therapy for hypoparathyroidism, comprising active vitamin D and often large doses of calcium, targets hypocalcemia and its acute symptoms but does not address the physiological consequences of insufficient PTH, including abnormally low bone remodeling.^15–18^ Other limitations of conventional therapy include high pill burden, large fluctuations in serum calcium, renal complications, and failure to improve quality of life.^19^ An ideal PTH therapy for hypoparathyroidism should provide PTH levels within the physiological range and restore endogenous calcitriol, thereby normalizing serum and urine biochemistry, and skeletal health, while promoting independence from conventional therapy.

Palopegteriparatide (TransCon PTH) is a prodrug of PTH (1-34), administered once daily, that provides active PTH within the physiological range for 24 hours/day.^20^ Palopegteriparatide has received regulatory approval in the US, EU, and several other countries.^21–23^ In the phase 2 PaTH Forward trial, adults with chronic hypoparathyroidism treated with palopegteriparatide achieved normal mean serum calcium, mean serum phosphate, and median urine calcium, and improved health-related quality of life over 26 weeks of treatment. Importantly, 91% of participants treated with palopegteriparatide also discontinued active vitamin D and therapeutic doses of calcium (defined as >500 mg/d) by week 26 of the PaTH Forward trial.^24^ The phase 3 PaTHway trial in adults with chronic hypoparathyroidism confirmed these benefits of palopegteriparatide treatment.^25^ Palopegteriparatide was well tolerated by the participants of both trials.

The PaTH Forward trial also showed that palopegteriparatide increases serum bone turnover markers (BTMs) from the low-normal to the mid-normal range.^24^ The bone resorption marker serum C-terminal telopeptide of type I collagen (CTx) increased from pre-treatment baseline, with peak mean levels at week 12 followed by a slight decline at week 26. The bone formation marker serum procollagen type 1 N-terminal propeptide (P1NP) also increased with palopegteriparatide treatment, reaching maximum levels at week 26. These findings suggest that palopegteriparatide enhances skeletal participation in calcium homeostasis by increasing calcium flux between blood and bone via the bone remodeling transient. The bone remodeling transient describes the temporary reduction in bone mass within a bone remodeling unit caused by the initial calcium-mobilizing resorptive phase of the remodeling cycle, which lasts for 1-2 months.^26^ This reduction in bone mass is followed by subsequent osteoblast-mediated refilling of resorption spaces with new mineralizing bone matrix, a process that typically lasts around 6-9 months in humans,^26^ with full mineralization of remodeling units taking up to 2-5 years.^27^ Here, we report longitudinal changes in BMD and BTMs in participants from the PaTH Forward trial through week 162. The effects of palopegteriparatide treatment on serum and urine biochemistries, independence from conventional therapy, and adverse events are also described.

## Materials and methods

### Trial design

**Figure 1:**
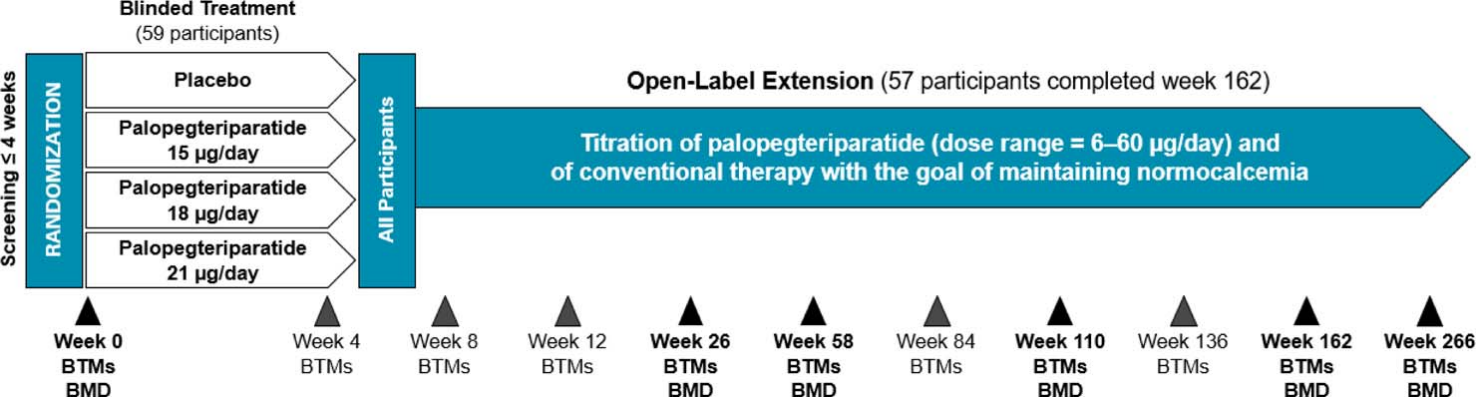
Design of the Phase 2 PaTH forward trial. After an initial 4-wk blinded, placebo-controlled treatment period, participants could enroll in an extension phase with open-label administration of palopegteriparatide, ending at wk 266. The current data reflect findings through wk 162. Black triangles identify trial visits where both BTM and BMD data were collected; gray triangles indicate BTMs only were collected. BTMs, bone turnover markers.

PaTH Forward (ClinicalTrials.gov: NCT04009291; EudraCT: 2018-004815-33) was trial with a 4-week randomized, double-blind, placebo-controlled period followed by an extension through week 266 that evaluated the efficacy, safety, and tolerability of palopegteriparatide in adults with chronic hypoparathyroidism (Figure 1). Data up to a prespecified week 162 interim analysis are presented herein. The protocol was reviewed by appropriate institutional review boards and ethics committees, and all participants provided signed informed consent prior to initiation. Additional details of the trial design were published previously.^24^

### Study population

Men and nonpregnant women aged ≥18 years with a body mass index of 17-40 kg/m^2^ and postsurgical, autoimmune, genetic, or idiopathic hypoparathyroidism were enrolled. Diagnosis of hypoparathyroidism was based on hypocalcemia with inappropriately low serum PTH for at least 26 weeks. According to the protocol, all participants were required to be taking stable doses of conventional therapy (active vitamin D defined as calcitriol ≥0.5 μg/d or alfacalcidol ≥1.0 μg/d; elemental calcium ≥800 mg/d) for at least 12 weeks before screening, and an estimated glomerular filtration rate ≥30 mL/min/1.73 m^2^ was also required. Thyroid-stimulating hormone (TSH) levels were required to be within normal laboratory limits in the 12 weeks prior to the first visit. Participants on suppressive therapy for thyroid cancer were required to have TSH levels ≥0.2 μIU/mL. Exclusion criteria included mutations in the calcium-sensing receptor gene, pseudohypoparathyroidism, diseases other than hypoparathyroidism that affect calcium or PTH homeostasis, and other significant morbidities. Also excluded were individuals taking certain medications (besides calcium and active vitamin D) known to influence calcium and bone metabolism, including loop or thiazide diuretics, phosphate binders, lithium, bisphosphonates, and PTH-like drugs.

### Trial protocol

Protocol details for screening, randomization, and the 4-week placebo-controlled fixed-dosing treatment period have been described previously.^24^ Individual conventional therapy regimens were optimized during screening to achieve serum 25OHD levels between 30 and 70 ng/mL and serum calcium within the normal range, which for this trial was 8.3-10.6 mg/dL (2.07-2.64 mmol/L) for albumin-adjusted serum calcium and 1.16-1.32 mmol/L for blood ionized calcium. During the placebo-controlled phase, participants were randomized to receive palopegteriparatide at 15, 18, or 21 μg PTH (1-34) or placebo via daily subcutaneous injection. Based on a predefined algorithm,^24^ participants with serum calcium within the normal range at prespecified visits had their active vitamin D progressively reduced until it could be discontinued, followed by reduced dosing of therapeutic calcium.

During the open-label dose titration period, participants previously treated with placebo or palopegteriparatide received daily palopegteriparatide as participants in the “all palopegteriparatide” group, with treatment individualized based on the continued need for active vitamin D. Those no longer needing active vitamin D continued on the same palopegteriparatide dose as previously, while those still receiving active vitamin D were started on a palopegteriparatide dose of 15 μg/day, with titration of active vitamin D and calcium as per the initial algorithm. Palopegteriparatide was increased by 3 μg/day for participants with calcium below the lower limit of normal or symptoms associated with hypocalcemia. Palopegteriparatide was decreased by 3 μg/day for participants with hypercalcemia who were no longer taking active vitamin D or calcium; absent those scenarios, palopegteriparatide was maintained at the same dose. Palopegteriparatide doses for the extension period ranged from 6-60 μg/day, and rescue doses of active vitamin D and/or calcium were allowed throughout.

### Efficacy assessments

Skeletal dynamics endpoints were evaluated as exploratory endpoints, including longitudinal assessments of BMD by DXA at the LS (L1-L4), TH, FN, and 1/3 distal radius, and the bone resorption marker serum CTx and the bone formation marker serum P1NP. Analysis and quality control of the blinded DXA scans were performed by a central facility (Clario).

Key efficacy endpoints during the extension period were serum calcium, 24-hour urine calcium, and independence from conventional therapy, defined as no active vitamin D and no more than 600 mg/day of elemental calcium.

### Safety assessments

Safety assessments performed at predefined intervals include serum and urine chemistries. Treatment-emergent adverse events (TEAEs), serious TEAEs (SAEs), and treatment-related TEAEs were collected and assessed at clinic visits and on review of participant diaries.

### Statistical analyses

Descriptive statistics were calculated for demographics, baseline disease characteristics, study drug exposure, clinical measurements, and safety endpoints based on all patients who received at least one dose of palopegteriparatide. Observed BTM and BMD values, their absolute change, and their percentage change from baseline during the extension period were summarized by study visit. Subgroup analysis of BMD and BTMs were performed on selected prognostic factors including pre/post-menopausal status, sex, baseline BMD, and duration of hypoparathyroidism. Statistical analyses were performed with SAS Version 9.4.

## Results

### Baseline demographics and disease characteristics

All 59 participants from the fixed-dose, 4-week blinded period participated in the open-label extension. One participant originally randomized to placebo discontinued after week 8 of the trial for protocol violation, one participant originally randomized to palopegteriparatide (18 μg/d) withdrew from the trial due to personal reasons, and the other 57 participants continued through week 162. At baseline, the mean age ± SD was 50 ± 12 year (range, 25-76 yr), with 54 of 59 participants (92%) under the age of 65. Eighty-one percent of the participants were female, of whom 65% were premenopausal (Table 1). The most common cause of hypoparathyroidism was neck surgery (*n* = 47; 79.7%), followed by idiopathic (*n* = 11; 18.6%) and autoimmune etiologies (*n* = 1; 1.7%). The mean duration of hypoparathyroidism was 12 year (range, 1-39 yr). Other participant baseline characteristics were previously reported.^24^

**Table 1:** Baseline demographics and disease characteristics of trial participants.

	All Participants (*N* = 59)
**Mean age in yr (SD)**	49.8 (12.1)
**Number (%) below 65 yr of age**	54 (91.5)
**Sex at birth, female, n (%)**	48 (81.4)
**Premenopausal females, n/N (%)**	31/48 (64.6)
**Race, % White**	91.5
**Geographic Region, %**	
**North America**	64.4
**Europe**	35.6
**Cause of hypoparathyroidism, %**	
**Acquired from neck surgery**	79.7
**Autoimmune disease**	1.7
**Idiopathic disease**	18.6
**Duration of hypoparathyroidism (yr)**	
**Mean (SD)**	11.9 (9.2)
**Median (range)**	9 (1-39)
**Conventional therapy, mean (SD) total daily dose**	
**Calcium (mg)**	1909 (1271)
**Calcitriol (μg)^a^**	0.79
**Alfacalcidol (μg)^b^**	2.38

a 46 participants (78%) used calcitriol at baseline;

b 13 participants (22%) used alfacalcidol at baseline.

### Bone mineral density

Changes in absolute BMD (g/cm^2^) in all DXA regions were greatest during the first 26 weeks of treatment, with mean changes from baseline ranging from −0.01 (1/3 distal radius) to −0.07% (LS and FN) (Table 2). The subsequent 32-week treatment interval comprising weeks 26-58 showed lower percent reductions in BMD, particularly at the LS, TH, and FN. The 52-week treatment interval from weeks 58-110 also showed more modest BMD declines, which ranged from −0.01 to −0.02, depending on the skeletal site. BMD declines were similarly modest from week 110 to week 162, ranging from 0.00 to −0.02 depending on the site.

**Table 2:** DXA results including absolute BMD and BMD T-scores and Z-scores.

	Overall population (*N* = 59)								
	Baseline Mean (SD)^a^	Wk 26 Mean (SD)^a^	Wk 58 Mean (SD)^a^	Wk 110 Mean (SD)^a^	Wk 162 Mean (SD)^a^	BMD Change Wk 0-26 Mean (SD)^b^	BMD Change Wk 26-58 Mean (SD)^b^	BMD Change Wk 58-110 Mean (SD)^b^	BMD Change Wk 110-162 Mean (SD)^b^
**Lumbar spine**									
***N***	57	46	46	55	55	46	47	47	57
**BMD (g/cm** ^**2**^ **)**	1.25 (0.23)	1.19 (0.22)	1.18 (0.21)	1.13 (0.20)	1.11 (0.21)	−0.07 (0.05)	−0.01 (0.05)	−0.02 (0.03)	−0.02 (0.03)
**Z-Score**	1.67 (1.68)	1.01 (1.62)	0.95 (1.54)	0.76 (1.43)	0.65 (1.49)	−0.62 (0.46)	−0.05 (0.38)	−0.15 (0.27)	−0.11 (0.29)
**T-Score**	1.04 (1.83)	0.39 (1.77)	0.32 (1.68)	0.03 (1.59)	−0.12 (1.66)	−0.64 (0.47)	−0.08 (0.39)	−0.20 (0.27)	−0.16 (0.30)
**Total hip**									
**N**	57	46	46	55	55	46	47	47	57
**BMD (g/cm** ^**2**^ **)**	1.05 (0.17)	1.02 (0.18)	1.00 (0.16)	0.97 (0.16)	0.95 (0.16)	−0.06 (0.05)	−0.02 (0.03)	−0.02 (0.03)	−0.02 (0.02)
**Z-Score**	0.98 (1.17)	0.60 (1.15)	0.53 (1.05)	0.37 (1.05)	0.27 (1.05)	−0.44 (0.37)	−0.10 (0.24)	−0.11 (0.26)	−0.09 (0.19)
**T-Score**	0.46 (1.26)	0.07 (1.29)	−0.02 (1.15)	−0.23 (1.17)	−0.36 (1.18)	−0.45 (0.38)	−0.12 (0.23)	−0.14 (0.26)	−0.13 (0.19)
**Femoral neck**									
**N**	57	46	46	55	55	46	47	47	57
**BMD (g/cm** ^**2**^ **)**	1.00 (0.19)	0.96 (0.19)	0.94 (0.18)	0.90 (0.18)	0.88 (0.18)	−0.07 (0.05)	−0.02 (0.04)	−0.02 (0.04)	−0.02 (0.03)
**Z-Score**	0.99 (1.15)	0.56 (1.16)	0.47 (1.05)	0.34 (0.99)	0.21 (1.0)	−0.48 (0.36)	−0.12 (0.30)	−0.10 (0.32)	−0.13 (0.21)
**T-Score**	0.20 (1.22)	−0.25 (1.29)	−0.36 (1.14)	−0.54 (1.11)	−0.71 (1.14)	−0.50 (0.36)	−0.14 (0.30)	−0.13 (0.32)	−0.17 (0.21)
**1/3 distal radius**									
**N**	55	43	43	53	52	43	45	46	56
**BMD (g/cm** ^**2**^ **)**	0.80 (0.14)	0.82 (0.12)	0.82 (0.11)	0.78 (0.14)	0.78 (0.13)	−0.01 (0.06)	−0.01 (0.03)	−0.01 (0.03)	−0.00 (0.02)
**Z-Score**	0.38 (1.31)	0.36 (1.04)	0.33 (1.01)	0.19 (1.14)	0.24 (1.16)	−0.07 (0.70)	−0.03 (0.33)	−0.06 (0.33)	0.01 (0.31)
**T-Score**	−0.22 (1.34)	−0.20 (1.16)	−0.21 (1.08)	−0.52 (1.30)	−0.51 (1.34)	−0.09 (0.70)	−0.06 (0.34)	−0.10 (0.34)	−0.03 (0.31)

a Statistical summaries are computed for all participants with data at baseline and the corresponding post-baseline visit.

b Data from participants with both corresponding visit values available were used to compute the statistical summaries for BMD change.

Mean BMD Z-scores at baseline were above zero for all DXA regions, indicating an age- and sex-adjusted excess in bone mass that is typical of chronic hypoparathyroidism (Table 2). Mean Z-scores for the LS, TH, and FN trended toward normalization (ie, 0.0) at week 26, followed by modest reductions through week 162. Mean (SD) Z-scores at week 162 remained above normal, with values of 0.65 (1.49) for the LS, 0.27 (1.05) for the TH, and 0.21 (1.0) for the FN. Mean Z-scores at the 1/3 distal radius were closer to zero at baseline compared with the other sites and showed more modest reductions over time, with the group mean of 0.24 (1.16) remaining above normal at week 162.

Mean BMD T-scores at baseline were above zero at the LS, TH, and FN (Table 2), signifying greater BMD than reference values for adults in the age range of peak bone mass. The overall patterns of T-score changes during treatment resembled those described above for Z-scores, with T-scores decreasing at the LS, TH, and FN primarily during the first 26 weeks, with a slower rate of decline thereafter. At week 162, mean BMD T-scores ranged from −0.12 to −0.71, well above the osteoporosis threshold of −2.5 and above the threshold of −1.0 that defines osteopenia.

**Figure 2:**
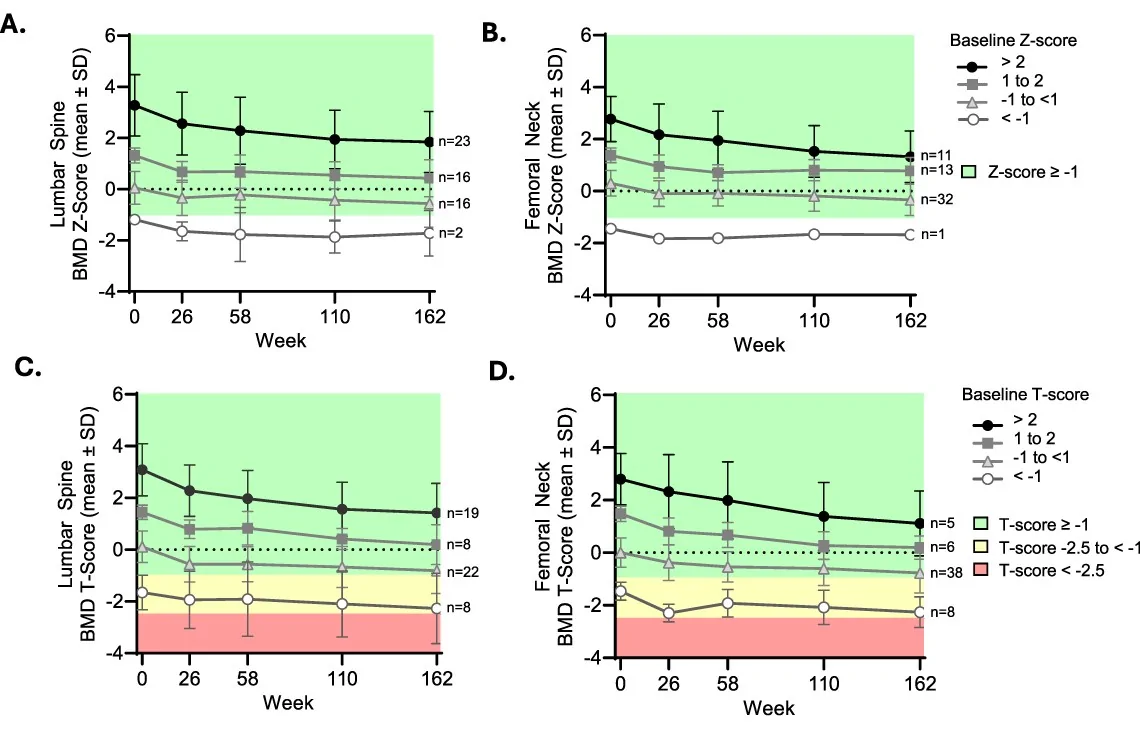
BMD through wk 162 by baseline BMD category <−1, −1, to <1, 1 to 2, and >2. (A) LS Z-scores, (B) FN Z-scores, (C) LS T-scores, and (D) FN T-scores. Data represent group means and SDs. Z-scores were based on age- and sex-matched reference ranges from the World Health Organization (WHO) and T-scores were based on data from young (30-yr-old) Caucasian adults in the WHO dataset.^45^

Exploratory analyses were performed to examine the inter-related hypotheses that participants with the highest baseline BMD and longest duration of hypoparathyroidism show the greatest skeletal calcium mobilization from baseline to week 162 as indicated by BMD decline. This entailed subgroup analyses of participants with baseline LS and FN BMD Z- and T-scores of <−1, −1, to <1, 1 to 2, and >2. In the subgroup with highest baseline Z-scores (>2), the change from baseline in LS and FN Z-scores was −1.43 (*n* = 23) and −1.45 (*n* = 11), respectively, compared with changes of −0.54 (*n* = 2) and −0.23 (*n* = 1), respectively, in the subgroup with the lowest baseline Z-scores (<−1) (Figure 2). Similarly, the subgroup with the highest baseline T-scores showed changes from baseline in LS and FN T-scores of −1.66 (*n* = 19) and −1.69 (*n* = 5), respectively, versus changes of −0.62 (*n* = 8) and −0.80 (*n* = 8), respectively, in the subgroup with the lowest baseline T-scores. The 2 subgroups with intermediate BMD Z- and T-scores at baseline generally showed intermediate changes in Z- and T-scores from baseline (Figure 2).

As expected, mean baseline and on-treatment Z-scores tended to be higher in participants with a longer duration of hypoparathyroidism (ie, >10 yr), particularly at the LS, TH, and FN (Figure S1). Duration of hypoparathyroidism was moderately related to BMD Z-score changes at the LS, TH, and FN, with a greater mean LS Z-score change from baseline to week 162 in the longest-duration group (−1.34) vs the shortest-duration group (−0.49). Mean Z-scores for the LS, TH, and FN regions remained at or above zero in each subgroup regardless of the duration of hypoparathyroidism. The BMD Z-score in the 1/3 distal radius region of all three subgroups remained stable over time regardless of the duration of hypoparathyroidism.

**Figure 3:**
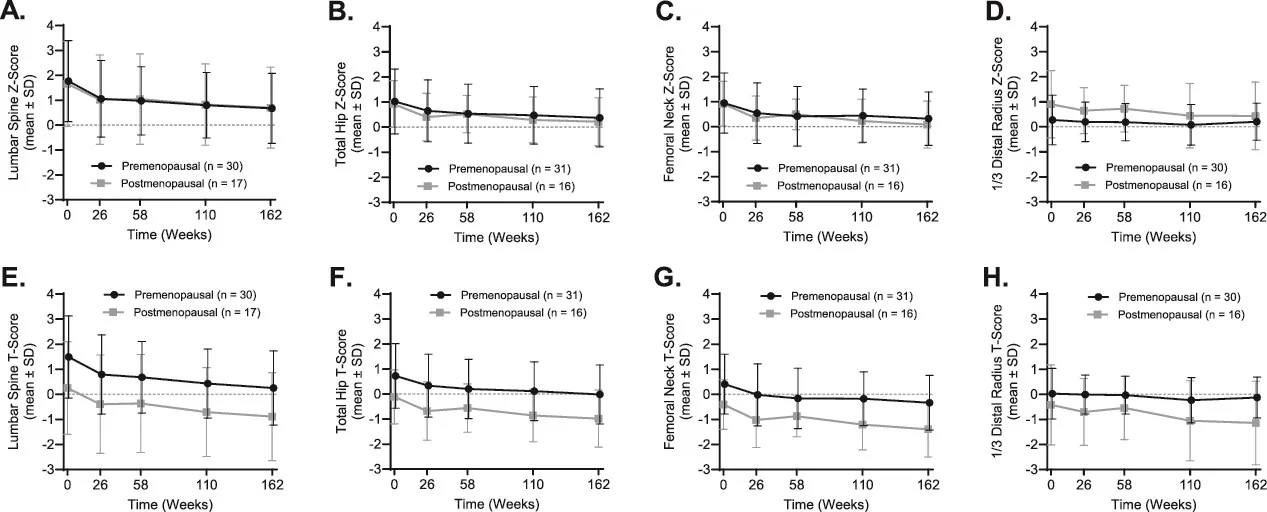
BMD T- and Z-scores according to menopausal status. BMD Z-scores (A-D) and T-scores (E-H) for the LS (A and E), TH (B and F), FN (C and G), and 1/3 distal radius (D and H) for female participants by baseline menopausal status. Data represent group means and SDs.

The patterns of BMD Z-score and T-score changes for female participants by baseline menopausal status are illustrated in Figure 3. As expected, mean baseline BMD T-scores tended to be relatively higher in the premenopausal subgroup, and mean T-scores in this subgroup remained well above −1.0 after 162 weeks of palopegteriparatide treatment. For postmenopausal women, mean baseline BMD T-scores were near zero for all sites and remained in the normal range (above −1.0) for the LS and TH, while decreasing to slightly below −1.0 for the FN and 1/3 distal radius. Mean BMD Z-scores remained above zero in premenopausal and postmenopausal women at all sites through 162 weeks of palopegteriparatide therapy.

For the 11 male participants, baseline mean BMD Z-scores were 1.30, 0.87, 1.20, and −0.29 at the LS, TH, FN, and 1/3 distal radius, respectively (Table S1). Mean Z-scores in these participants declined at the LS, TH, and FN to levels that remained above zero at week 162. Mean T-scores for all sites except the 1/3 distal radius were above zero at baseline, followed by decreases to levels that remained above −1.0 through week 162. The mean BMD of the 1/3 distal radius showed no net decrease from baseline to week 162.

### Bone turnover markers

**Figure 4:**
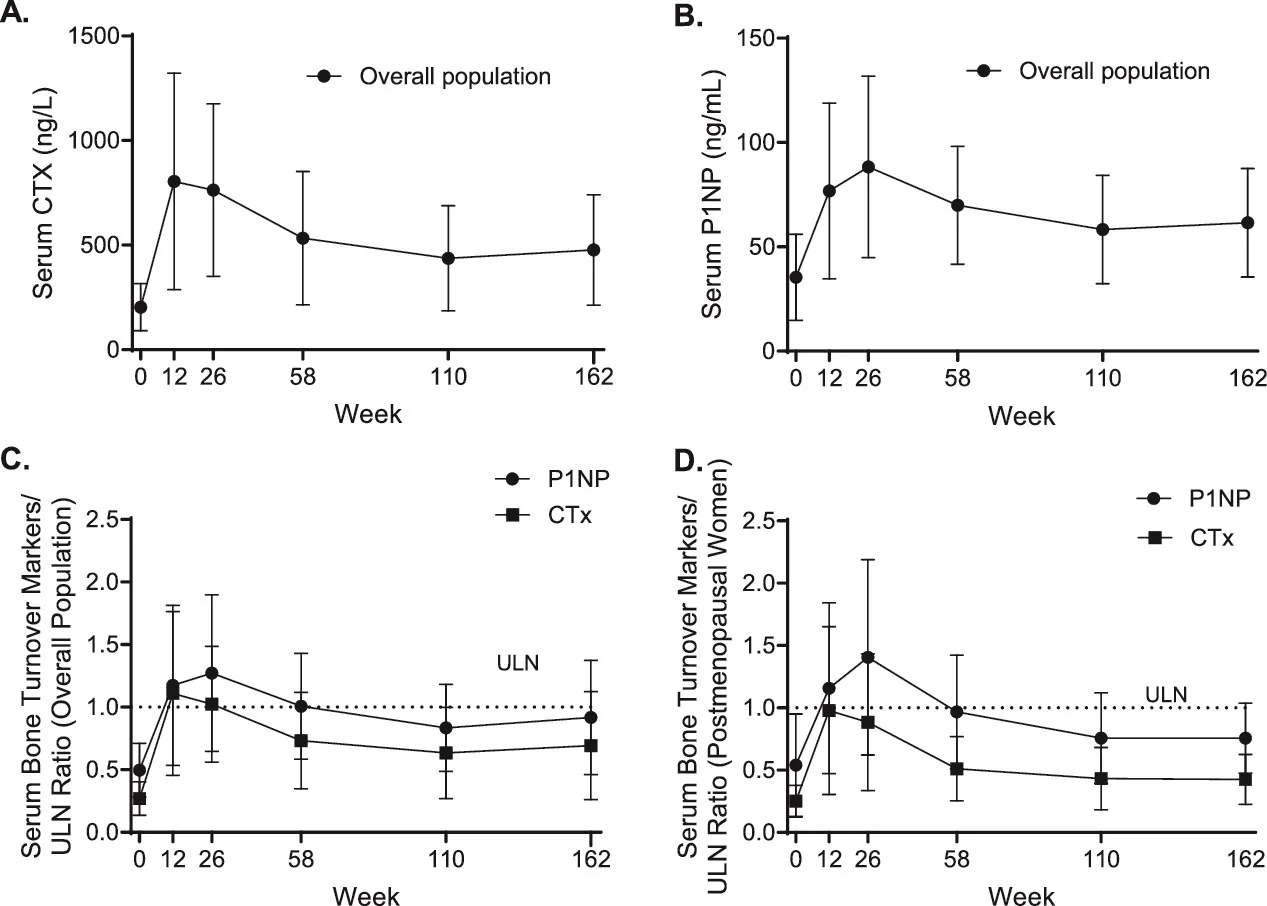
Serum bone turnover markers. (A and B) Serum C-terminal telopeptide of type 1 collagen (CTx) and serum procollagen type 1 N-terminal propeptide (P1NP) for the overall study population. (C and D) Serum CTx and serum P1NP for the overall population and the postmenopausal female subgroup as a proportion of the upper limit of normal (ULN). Data represent group means and SDs.

Trends in the bone resorption marker CTx and the bone formation marker P1NP were consistent with the observed changes in BMD from the DXA scans. A coupled bone formation and resorption response was observed with mean biomarker levels peaking by week 26 (Figure 4, Figure S2). For the overall population, serum CTx increased from a mean (SD) of 202.8 (112.7) ng/L at baseline to a mean peak of 804.2 (517.2) ng/L at week 12 and then declined and remained stable above baseline levels from week 110 through week 162 with a mean (SD) value of 476.9 (263.6) ng/L at week 162 (Figure 4A). The bone formation marker serum P1NP increased from a mean (SD) of 35.4 (20.6) ng/mL at baseline to a mean (SD) peak of 88.4 (43.6) ng/mL at week 26, followed by a decline and stabilization above baseline to a mean (SD) of 61.5 (26.0) ng/mL at week 162 (Figure 4B). Figure 4C and D shows serum CTx and P1NP in the postmenopausal female subgroup expressed as a proportion of the upper limit of normal (ULN) for each BTM. For CTx, this ratio remained at or below 1.0 throughout the 162-week treatment period, indicating a relatively normal rate of bone resorption in this subgroup with the lowest BMD T-scores at baseline. The P1NP to ULN ratio in the postmenopausal female subgroup exceeded 1.0 at weeks 12 and 26, indicating a brief period of greater-than-normal bone formation. The ULN for CTx and P1NP in subpopulations of trial participants are included in Figure S2.

From weeks 58-162, mean serum CTx and P1NP levels for pre- and post-menopausal female and male trial participants were generally below the upper limits of their respective normal reference ranges, as highlighted on the graphs, with the exception of P1NP in premenopausal women at weeks 58 and 162 (Figure S2).

### Palopegteriparatide treatment compliance

Treatment compliance with palopegteriparatide therapy averaged 99.8% and exceeded 90% for every participant through week 162.

### Independence from conventional therapy

Overall, the response to treatment with palopegteriparatide was sustained throughout week 162 of the PaTH Forward trial. Fifty-six (56) of 57 participants (98%) who continued through week 162 achieved independence from active vitamin D therapy (ie, 0 μg/d). Fifty-two (52) participants (91%) achieved independence from both active vitamin D and therapeutic doses of calcium (ie, ≤600 mg/d).

### Serum and urine calcium

**Figure 5:**
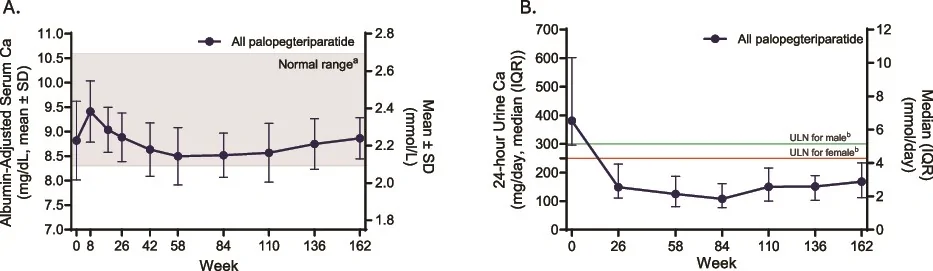
Serum and urine calcium. (A) Mean albumin-adjusted serum calcium (Ca) and (B) median 24-h urine calcium results. The shaded area in (A) represents the normal serum calcium range of 8.3-10.6 mg/dL. In (B), the upper limit of normal (ULN) for men and women are depicted by horizontal lines. Error bars reflect SDs for serum calcium and interquartile range for urine calcium. *N* = 52-59 for serum calcium and 49-54 for urine calcium.

With palopegteriparatide treatment, mean serum calcium levels were maintained within the normal range through week 162 and demonstrated the durability of response to palopegteriparatide treatment over time in PaTH Forward trial participants (Figure 5A). At baseline, 24-hour median urine calcium was 381 mg/d, which is above the ULN for men (300 mg/d) and women (250 mg/d) (Figure 5B). Median urine calcium levels normalized by week 26 and were maintained below 250 mg/d through week 162.

### Safety assessment

Treatment-emergent adverse events were reported by 57/59 (97%) participants. The majority of TEAEs were mild (Grade 1) or moderate (Grade 2) and most were unrelated to the study drug. There were no treatment-related SAEs and no TEAEs that led to treatment discontinuation of treatment or the trial through week 162. No events of hypercalcemia or hypocalcemia leading to an emergency room or urgent care visit or hospitalization occurred during the open-label extension period. To date, there have been 8 events of fractures occurring in 6 participants, all assessed by the investigator as unrelated to the study drug. These fractures included one fragility fracture, which was a rib fracture from a fall from standing height. Additional sites of fracture were vertebra, tibia, metacarpal, phalanx, navicular bone, and metatarsal.

## Discussion

These 162-week data from the phase 2 PaTH Forward trial show continued PTH replacement therapy with palopegteriparatide resulted in sustained temporal changes trending toward a new skeletal steady state, closer to age-appropriate norms. Baseline BMD Z-scores at the LS, TH, and FN indicate that initial bone mass at these sites was between 1 and 1.6 SDs above age- and sex-matched reference ranges. With palopegteriparatide treatment, mean BMD Z-scores at the LS, TH, and FN trended toward age- and sex-matched norms through week 162. Mean BMD T-scores remained above 1.0 at all sites through week 162. The 1/3 distal radius, a predominantly cortical site, showed the smallest excess in BMD at baseline and the smallest treatment-related decrease in BMD. At later timepoints, mean FN and 1/3 distal radius BMD T-scores among postmenopausal women were slightly below the osteopenia threshold at weeks 110 and 162. Modest declines in FN and distal radius BMD are also observed during daily teriparatide therapy in women with postmenopausal osteoporosis, but these changes were not associated with reductions in bone structure or bone strength parameters,^28^^,^^29^ and may be associated with a biomechanical advantage by increasing the cross-sectional area of the bone.^30^^,^^31^ At the LS, TH, and FN, lower baseline BMD Z- and T-scores were associated with smaller declines in BMD over time, suggesting that bone loss is attenuated in patients who may be considered more vulnerable to fracture. Baseline and on-study BMD Z-scores were generally higher in participants with a longer duration of hypoparathyroidism, potentially reflecting a longer period of reduced bone remodeling prior to initiation of treatment with palopegteriparatide.

Changes in serum biomarkers of bone remodeling reflected the longitudinal changes in BMD, as shown by early increases in mean serum CTx and P1NP levels that progressively attenuated over time to levels that remained above baseline at week 162. Consistent with this bone remodeling profile, palopegteriparatide treatment led to early decreases in BMD at week 26 followed by a slower decline toward age- and sex-matched BMD levels over the subsequent 84 weeks of treatment. Bone remodeling is an important contributor to calcium homeostasis by increasing skeletal calcium release to serve metabolic needs, an effect which temporarily reduces bone mass via the remodeling transient.^26^ Individuals with hypoparathyroidism have a tendency toward higher bone mass due to reduced bone remodeling and higher bone matrix mineralization.^5^ Reduced bone remodeling can also lead to challenges in maintaining blood calcium within a narrow normal range.^4^^,^^32^

Treatment with palopegteriparatide resulted in an initial rise in the resorption marker serum CTx through week 26, which then decreased at weeks 110-162 to levels that suggest attainment of a steady state within normal reference ranges. An increase in bone resorption markers with subsequent attenuation is a typical response to treatment with PTH receptor agonists, including agents and regimens used for hypoparathyroidism^8^^,^^33^^,^^34^ and osteoporosis.^35^ The attenuated CTx response from weeks 110 through 162 occurred despite slight increases in the average daily palopegteriparatide dose compared with week 12 when serum CTx was maximal. When accompanied by a reduced rate of bone loss, the still elevated remodeling rate is consistent with a restoration of the PTH axis at a new steady state. Evidence that the mean blood calcium levels remained within the normal range despite substantial attenuation of the initial CTx response and the reduced rate of BMD decline suggests that the longer-term efficacy of palopegteriparatide relates to additional contributions from non-skeletal calcium regulatory mechanisms such as enhanced renal and intestinal calcium absorption.

Serum bone formation marker P1NP increased to its maximum mean level at week 26, followed by a decrease through week 110 and stabilization thereafter. An early increase in P1NP followed by gradual attenuation of that response is observed with most PTH receptor agonist therapies, including PTH (1-84).^6^^,^^8^^,^^33–35^ PTH (1-84) therapy in individuals with hypoparathyroidism has generally neutral or positive effects on BMD,^8^^,^^36^ suggesting that this treatment, which creates brief daily increases in PTH (1-84) levels, causes minimal net skeletal calcium mobilization into the blood. Conversely, the current BMD data with palopegteriparatide suggest that this prodrug, which provides active PTH within the physiological range for 24 hours/day,^20^ leads to a net increase in skeletal calcium mobilization that contributes to the maintenance of blood calcium subsequently fostering independence from conventional therapy. We speculate that unlike intermittent PTH regimens that predominantly stimulate modeling-based bone formation,^37^^,^^38^ the sustained physiological PTH levels achieved with palopegteriparatide might optimize the balance between remodeling-based calcium mobilization and modeling-based structural enhancement, potentially improving bone quality while maintaining skeletal participation in calcium homeostasis. In addition, the absence of a net bone anabolic effect with palopegteriparatide reduces the theoretical increase in risk of osteosarcoma, which has been reported in rats after daily administration of PTH resulted in increased bone mass.^39^^,^^40^

The implications for potential fracture risk of both the elevated baseline BMD characteristic of hypoparathyroidism and treatment-related changes are not well established; however, several observations suggest that such risk would be minimal. First, mean serum CTx values rarely exceeded the ULN, with the exception of premenopausal women at week 12 and 26, at which time their BMD T-scores were at or above 0. Serum P1NP increased transiently above the ULN in pre- and post-menopausal women, which may reflect robust coupling within remodeling units as they refill resorption spaces. Secondly, participants with the lowest baseline BMD Z- and T-scores tended to experience smaller BMD changes compared with participants with the highest Z- and T-scores. Thirdly, the nadir of BMD at weeks 110-162 remained above expected levels and did not coincide with maximal BTM responses. Mechanistically, one hypothesis is that increased bone remodeling with palopegteriparatide could mitigate fracture risk by renewing bone matrix that tends to be hypermineralized in individuals with chronic hypoparathyroidism.^1^^,^^9^ Consistent with this hypothesis, reactivation of bone remodeling with daily teriparatide was shown to decrease parameters of hypermineralization in paired bone biopsies taken from patients with hypoparathyroidism.^9^ Although fractures were reported in this study, most events represented trauma-related peripheral fractures rather than fragility fractures, and impact of palopegteriparatide on the occurrence of fractures would require evaluation in larger, longer-term studies.

Palopegteriparatide treatment through week 162 fulfilled key hypoparathyroidism treatment goals, including the maintenance of serum calcium within the lower range of normal without conventional therapy.^1^^,^^18^^,^^41^ Another important treatment goal for hypoparathyroidism is the reduction of elevated urine calcium, which may mitigate the risk of renal complications.^2^^,^^41^ Palopegteriparatide treatment normalized urine calcium, with persistent reductions from baseline of ≥60% from week 26 to week 162. Palopegteriparatide continued to be well tolerated and associated with a favorable safety profile. The majority of TEAEs were mild or moderate and unrelated to the study drug. There were no serious treatment-related TEAEs, and no participants discontinued the trial due to TEAEs.

Strengths of the trial include individualized titrated palopegteriparatide dosing during the open-label extension period and allowance of rescue doses of conventional therapy, which may reflect elements of real-world management of hypoparathyroidism, and the high participation rate in the extension (57 of 59 participants). Several limitations warrant consideration, including the lack of bone biopsy histomorphometry, which represents the gold standard for evaluating tissue-level bone remodeling. Previous histomorphometry data for individuals with hypoparathyroidism indicate markedly reduced bone remodeling, even though serum BTMs were only reduced to the lower half of the normal range,^32^ similar to the BTM profile in the current trial. The lack of special imaging modalities, such as quantitative CT, precludes the evaluation of changes in bone microstructure, geometry, or biomechanical indices. Blood was collected without a fasting requirement to limit the risk of excursions in blood calcium, which may have impacted some analytes including CTx. Finally, our cohort included mostly white women with small numbers of men and other racial groups.

In summary, these analyses from the open-label extension of the phase 2 PaTH Forward trial in adults with chronic hypoparathyroidism demonstrate that treatment with palopegteriparatide for up to 162 weeks normalized both BMD from characteristically elevated levels and bone turnover toward age-appropriate physiological ranges. Treatment with palopegteriparatide also maintained blood calcium in the normal range and reversed hypercalciuria, with 91% of participants achieving independence from conventional therapy. Palopegteriparatide remained well tolerated through week 162, with no new safety findings compared with the initial treatment phase.^24^ The sustained release of PTH from palopegteriparatide distinguishes it from the high daily peak-to-trough pharmacokinetic profiles of short-lived PTH receptor agonists, including PTH (1-84),^42^ PTH (1-34),^43^ and eneboparatide (LA-PTH, AZP-3601).^44^ We hypothesize that the sustained PTH exposure profile contributes to palopegteriparatide’s ability to maintain blood calcium without conventional therapy by initially increasing remodeling-based skeletal calcium mobilization, which attenuates over time, plus potential contributions from other calcium homeostatic pathways in extraskeletal tissues such as the intestine and the kidney. The results of the long-term open-label extension periods of both the phase 2 PaTH Forward and phase 3 PaTHway trials will further characterize the longer-term effects of palopegteriparatide on bone and other parameters.

## Supplementary Material

Supplemental_Figure_1_zjag013

Supplemental_Figure_2_zjag013

Supplemental_Table_1_zjag013

CONSORT_Consolidated_Standards_of_Reporting_Trials_zjag013

## Data Availability

The datasets generated during and/or analyzed during the present study are not publicly available but are available from the corresponding author on reasonable request.

## Study summary

This analysis examined the long-term effects of daily palopegteriparatide treatment on skeletal dynamics in adults with chronic hypoparathyroidism through year 3 of the phase 2 PaTH Forward trial.

## Notes

ClinicalTrials.gov registration number NCT04009291; EudraCT number 2018-004815-33.
