# Supplementary material for: Palopegteriparatide for adults with chronic hypoparathyroidism: skeletal dynamics through 3 years of the phase 2 PaTH forward trial
Source: J Bone Miner Res. 2026 Feb 4;41(10):1069–79. doi: 10.1093/jbmr/zjag013 (PMC13616104; doi:10.1093/jbmr/zjag013)
Supplement: Supplementary file 1 [file consort_consolidated_standards_of_reporting_trials_zjag013.pdf]

JBMR®

2001 K Street, NW,
3rd Floor North
Washington, DC
20006, USA

P: 202-367-2336
E: jbmr@asbmr.org

Corresponding Author Name: Aimee D Shu

Manuscript Title: Palopegteriparatide for Adults with Chronic Hypoparathyroidism: Skelet

Randomized Clinical Trials: Consolidated Standards of Reporting Trials Checklist

When reporting results of clinical trials, the *JBMR*® adheres to the CONSORT statement , and authors must provide the information required by the CONSORT checklist, as well as a flow diagram in CONSORT format, when they submit their manuscript. This material may be provided as supplemental information in the event that its inclusion exceeds the page limit for the manuscript. Since statistical analyses vary according to study design and prohibit the establishment of standard rules, the following guidelines are provided to assist authors in performing the most appropriate statistical analyses and reporting those results in accord with *JBMR*® standards. If you have any questions, please contact us at jbmr@asbmr.org.

Journal of Bone and Mineral Research
JBMR®

Recommendation		Page
Title/Abstract-		1, 6
Identification as a randomized trial in the title<br>Structured summary of trial design, methods, results, and conclusions (for specific guidance see CONSORT for abstracts)		
Introduction-		
Scientific background and explanation of rationale		8
Specific objectives or hypotheses		9
Methods-		
Trial design	Description of trial design (such as parallel, factorial) including allocation ratio	9
	Important changes to methods after trial commencement (such as eligibility criteria), with reasons	10
Participants	Eligibility criteria for participants	10
	Settings and locations where the data were collected	10
Interventions	The interventions for each group with sufficient details to allow replication, including how and when they were actually administered	10
Outcomes	Completely defined pre-specified primary and secondary outcome measures, including how and when they were assessed	11
	Any changes to trial outcomes after the trial commenced, with reasons	NA
Sample size	How sample size was determined	12
	When applicable, explanation of any interim analyses and stopping guidelines	12
Methods- Randomization		
Sequence generation	Method used to generate the random allocation sequence	10
	Type of randomization; details of any restriction (such as blocking and block size)	10

Recommendation		Page
Methods cont'd		NA
Allocation concealment mechanism	Mechanism used to implement the random allocation sequence (such as sequentially numbered containers), describing any steps taken to conceal the sequence until interventions were assigned	
Implementation	Who generated the random allocation sequence, who enrolled participants, and who assigned participants to interventions	9-10
Blinding	If done, who was blinded after assignment to interventions (for example, participants, care providers, those assessing outcomes) and how	9
	If relevant, description of the similarity of interventions	NA
Statistical methods	Statistical methods used to compare groups for primary and secondary outcomes	12
	Methods for additional analyses, such as subgroup analyses and adjusted analyses	12
Results-		26
Participant flow (a diagram is strongly recommended)	For each group, the numbers of participants who were randomly assigned, received intended treatment, and were analyzed for the primary outcome	
	For each group, losses and exclusions after randomization, together with reasons	12
Recruitment	Dates defining the periods of recruitment and follow-up	NA
	Why the trial ended or was stopped	NA
Baseline data	A table showing baseline demographic and clinical characteristics for each group	23
Numbers analyzed	For each group, number of participants (denominator) included in each analysis and whether the analysis was by original assigned groups	23-30
Outcomes and estimation	For each primary and secondary outcome, results for each group, and the estimated effect size and its precision (such as 95% confidence interval)	12-16
	For binary outcomes, presentation of both absolute and relative effect sizes is recommended	NA
Ancillary analyses	Results of any other analyses performed, including subgroup analyses and adjusted analyses, distinguishing pre-specified from exploratory	12-16
Harms	All important harms or unintended effects in each group (for specific guidance see CONSORT for harms)	16

Recommendation		Page
Discussion –		20
Limitations	Trial limitations, addressing sources of potential bias, imprecision, and, if relevant, multiplicity of analyses	
Generalizability	Generalizability (external validity, applicability) of the trial findings	17-21
Interpretation	Interpretation consistent with results, balancing benefits and harms, and considering other relevant evidence	17-21
Other Information-		3
Registration	Registration number and name of trial registry	
Protocol	Where the full trial protocol can be accessed, if available	NA
Funding	Sources of funding and other support (such as supply of drugs), role of funders	3
