# Supplementary material for: Palopegteriparatide for adults with chronic hypoparathyroidism: skeletal dynamics through 3 years of the phase 2 PaTH forward trial
Source: J Bone Miner Res. 2026 Feb 4;41(10):1069–79. doi: 10.1093/jbmr/zjag013 (PMC13616104; doi:10.1093/jbmr/zjag013)
Supplement: Supplementary file 2 [file supplemental_table_1_zjag013.docx]

**Supplemental Table 1:** DXA results for male participants.

|  | Men (N = 11) | | | | | |
| --- | --- | --- | --- | --- | --- | --- |
|  | Baseline Mean (SD) | Week 26 Mean (SD) | Week 58 Mean (SD) | Week 110 Mean (SD) | Week 162 Mean (SD) | Absolute Change  BL to Wk 162 (SD) |
| Lumbar Spine | | | | | | |
| N | 10 | 9 | 9 | 10 | 10 | 10 |
| BMD (g/cm^2^) | 1.26 (0.26) | 1.20 (0.24) | 1.19 (0.24) | 1.16 (0.21) | 1.16 (0.24) | -0.10 (0.14) |
| T-Score | 0.98 (2.01) | 0.41 (1.75) | 0.30 (1.72) | 0.10 (1.51) | 0.13 (1.70) | -0.85 (1.26) |
| Z-Score | 1.30 (1.89) | 0.78 (1.69) | 0.68 (1.67) | 0.48 (1.47) | 0.52 (1.65) | -0.78 (1.26) |
| Total Hip | | | | | | |
| N | 10 | 9 | 9 | 10 | 10 | 10 |
| BMD (g/cm^2^) | 1.14 (0.17) | 1.11 (0.18) | 1.08 (0.16) | 1.03 (0.14) | 1.02 (0.14) | -0.13 (0.07) |
| T-Score | 0.49 (1.14) | 0.28 (1.19) | 0.04 (1.06) | -0.28 (0.89) | -0.38 (0.89) | -0.87 (0.51) |
| Z-Score | 0.87 (1.14) | 0.70 (1.18) | 0.46 (1.07) | 0.15 (0.93) | 0.06 (0.91) | -0.81 (0.50) |
| Femoral Neck | | | | | | |
| N | 10 | 9 | 9 | 10 | 10 | 10 |
| BMD (g/cm^2^) | 1.08 (0.23) | 1.04 (0.22) | 0.98 (0.21) | 0.93 (0.18) | 0.91 (0.18) | -0.17 (0.09) |
| T-Score | 0.48 (1.37) | 0.09 (1.30) | -0.28 (1.15) | -0.58 (0.89) | -0.70 (0.89) | -1.18 (0.69) |
| Z-Score | 1.20 (1.36) | 0.85 (1.30) | 0.50 (1.14) | 0.19 (0.95) | 0.10 (0.94) | -1.10 (0.68) |
| 1/3 Distal Radius | | | | | | |
| N | 9 | 7 | 8 | 9 | 9 | 9 |
| BMD (g/cm^2^) | 0.84 (0.14) | 0.89 (0.14) | 0.86 (0.12) | 0.85 (0.14) | 0.85 (0.14) | 0.01 (0.04) |
| T-Score | -0.75 (1.71) | -0.12 (1.70) | -0.39 (1.55) | -0.54 (1.59) | -0.53 (1.68) | 0.21 (0.75) |
| Z-Score | -0.29 (1.82) | 0.34 (1.79) | 0.14 (1.65) | -0.01 (1.67) | 0.02 (1.78) | 0.30 (0.74) |

DXA, dual X-ray absorptiometry. BMD, bone mineral density. BL, baseline; Wk, week.
